# A Review on Dengue Vaccine Development

**DOI:** 10.3390/vaccines8010063

**Published:** 2020-02-02

**Authors:** Sheng-Qun Deng, Xian Yang, Yong Wei, Jia-Ting Chen, Xiao-Jun Wang, Hong-Juan Peng

**Affiliations:** 1 Department of Pathogen Biology, Guangdong Provincial Key Laboratory of Tropical Disease Research, School of Public Health, Southern Medical University, Guangzhou 510515, China; dengshengqun@163.com (S.-Q.D.); xianluebuzixian28@163.com (X.Y.); smuweiyong@163.com (Y.W.); jiating723@i.smu.edu.cn (J.-T.C.); 2 Department of Epidemiology and Biostatistics, School of Public Health, Guangdong Medical University, Dongguan 523808, China; wangxj1664@gdmu.edu.cn

**Keywords:** dengue virus, dengue fever, vaccine, vector-borne disease

## Abstract

Dengue virus (DENV) has become a global health threat with about half of the world’s population at risk of infection. Although the disease caused by DENV is self-limiting in the first infection, the antibody-dependent enhancement (ADE) effect increases the mortality in the second infection with a heterotypic virus. Since there is no specific efficient medicine in treatment, it is urgent to develop vaccines to prevent infection and disease progression. Currently, only a live attenuated vaccine, chimeric yellow fever 17D—tetravalent dengue vaccine (CYD-TDV), has been licensed for clinical use in some countries, and many candidate vaccines are still under research and development. This review discusses the progress, strengths, and weaknesses of the five types of vaccines including live attenuated vaccine, inactivated virus vaccine, recombinant subunit vaccine, viral vectored vaccine, and DNA vaccine.

## 1. Introduction

Dengue virus (DENV) is a positive-sense, single-stranded RNA (+ssRNA) virus belonging to genus Flavivirus of family Flaviviridae [1]. The mature particles of DENV are spherical and 50 nm in diameter [2]. The virus contains multiple copies of three structural proteins, including Envelope (E) protein, Membrane (M) protein, and Capsid (C) protein; a host-derived lipid bilayer envelope; and a single copy of RNA genome [2]. DENV has four serotypes (DENV 1–4) that are transmitted in humans by *Aedes* mosquitoes [1]. The most common clinical manifestations are sudden fever with headache, recurrent eyelid pain, generalized muscle pain and joint pain, blushing, anorexia, and abdominal pain. All four serotypes can cause dengue fever (DF), dengue hemorrhagic fever (DHF), and dengue shock syndrome (DSS) [3]. DHF and DSS are the more severe effects and more commonly seen in the infection of children and adolescents under fifteen years old [4]. DENV infection produces a high titer of neutralizing antibodies, which are considered an important component of the protective immune response [5,6]. Homotypic protection (protection against the same serotype infection) is considered long-term effective after a serotype infection, while heterotypic protection (cross protection against the other serotypes infection) can last for approximately two years [7,8]. With the reduction of cross-antibody titer, the second heterotypic dengue infection will be more serious than the first [9]. Furthermore, non-neutralizing antibodies can form complexes with DENV particles and can facilitate virus infection to phagocytic cells via Fc receptors, resulting in enhanced infection and leading to DHF and DSS. This phenomenon is called antibody-dependent enhancement (ADE) [3]. It is estimated that 390 million dengue infections happen every year, of which 96 million manifest clinically at any level of disease severity [10]. There is currently no specific medicine for dengue treatments, and prevention majorly relies on vector control. Therefore, dengue vaccine development is urgently required for dengue prevention.

Five types of dengue vaccines have been under investigation, including live attenuated vaccine, inactivated vaccine, recombinant subunit vaccine, viral vectored vaccine, and DNA vaccine [11]. They act primarily by increasing the immune responses against dengue virus (DENV) E protein and non-structural protein 1 (NS1) [12]. Careful studies of the immune responses to DENV help to form an effective strategy for dengue vaccine development [13].

Two major challenges in dengue vaccine development have been discussed. Firstly, although DENV antibodies show protective effects against homotypic or heterotypic DENV infection, the ADE effect resulting from a second heterotypic infection majorly accounts for DHF and DSS [14,15,16]. However, the immune response and pathogenesis of DHF and DSS are not fully understood, which hinders DENV vaccine development [17]. Secondly, in vaccine development, we lack a conveniently accessible, cheap, and sensitive animal model capable of simulating the immune responses in humans after infection. Since mice are naturally resistant to DENV infection, human cell chimeric mice and immunodeficient mice sensitive to DENV infection are established to be used as animal models [18]. Nonhuman primates (NHPs) are highly potential animal models because they produce a similar immune response to DENV infection as humans, but they are usually used following mouse tests because of the costliness [19].

## 2. Live Attenuated Vaccine

Live attenuated vaccines are antigenic substances composed of a living pathogen, but the pathogen is altered to be less virulent or avirulent [20]. Live attenuated vaccines show the advantages of delivering a set of protective antigens and of providing long-term immune protectivity [20]. Several live dengue attenuated vaccines have been made with recombinant DNA technology, such as the chimeric yellow fever 17D virus-tetravalent dengue vaccine (CYD-TDV), the recombinant DENV-4 mutant bearing a 30-nucleotide deletion vaccine (rDEN4∆30), and the tetra-live attenuated virus dengue vaccine (DENVax) [21].

### 2.1. Live Attenuated Chimeric Yellow Fever–Dengue Vaccines

The clinically developed dengue vaccine, CYD-TDV (Dengvaxia^®^) (Sanofi, Paris, France), complying with the International Guidelines for New Vaccines [22] has been licensed by several dengue-endemic countries in Asia and Latin America for use in people over 9 years old [23]. This vaccine was constructed by replacing the prM/E RNAs of the YF17D (yellow fever virus vaccine strain) with the corresponding sequences of the four dengue serotypes [24]. It has been observed in clinical trials that vaccination with CYD-TDV is more effective among people over 9 years old [25]. The vaccine-stimulated immunity lasts up to 4 years, and the virus serotype, age, and dengue sera status of the individual before vaccination seem to affect the vaccine effectiveness [23]. However, long-term safety assessments showed that the risk of hospitalization for vaccinees was greater than that of placebo controls 3 years after vaccination [26]. The reason for the reduced protectivity of CYD-TDV in seronegative subjects and the increased risk of hospitalization for children under 9 years old is unclear [27]. The CYD-TDV IIb clinical trial in Thailand and phase III clinical trials in Asia and Latin America found that it was ineffective against DENV2 and that the first-time immunization was less effective than re-immunization [25,28,29]. The better efficacy of re-immunization may be due to the enhanced protectivity in the subjects by stimulating preexisting immune memory [30]. In April 2018, the WHO Strategic Advisory Panel recommended that, in CYD-TDV vaccination, the priority assessment of DENV serostatus should be considered to ensure that only dengue-seropositive individuals are vaccinated because CYD-TDV vaccination in seronegative subjects increases the risk of severe dengue [31]. Therefore, rapid diagnostic tests are needed before the vaccination. Since DENV belongs to the genus Flavivirus which also includes Zika virus, yellow fever virus, and tick-borne encephalitis virus with highly homologous genomes, proteins, and antibodies, antibody detection for these virus infections has high cross-reactivity and poor reliability [32]. The plaque reduction neutralization test (PRNT) is the gold standard for establishing serum status but requires specific laboratory and technical capabilities. Furthermore, the detection process of dengue IgG ELISA is complicated, and the diagnosis of IgG-containing point-of-care tests lack sensitivity and specificity. Therefore, platform evaluation and well-characterized vaccine samples are required to accelerate the clinical trials and market entry for CYD-TDV [33].

### 2.2. Live Attenuated rDEN∆30 Vaccines

There have been some studies to integrate DENV monovalent vaccines with the live attenuated tetravalent vaccine (LATV) [34]. However, it is a challenge to develop a LATV that not only has immunogenicity for all four dengue serotypes but also has sufficient attenuation of each monovalent component [35,36,37]. The 3′- UTR region of the flavivirus genome was chosen as a target for mutagenesis because it plays an important role in viral RNA replication [38]. The LATV rDEN4∆30 vaccination stimulated a seven-fold or greater increase in serum neutralizing antibody titer (mean titer = 1:580) on day 28 post-vaccination, and was found to be very tolerant to all of the vaccinees, in which only a mild, asymptomatic macular rash developed, and a transient elevation in the serum level of alanine aminotransferase was noted in several volunteers [39]. Prior to this, the experiments testing the ability of the vaccine virus spreading from vaccinees to mosquito vectors showed that no vaccine virus was found in all 352 experimental mosquitoes [40]. Moreover, in order to increase the decay rate of rDEN4∆30 and to reduce the side effects, many studies have optimized the paired charge–mutation of alanine and obtained rDEN-4∆30NS5-K200A and rDEN-4∆30NS5-H201A, which show good compatibility in human vaccination [41,42,43]. Researchers also constructed rDEN4∆30 through chemical mutagenesis and successfully obtained rDEN-4∆30 NS3-S158R [41,44].

TV003 is a mixture of four attenuated recombinant dengue vaccine candidates including rDEN1D30, rDEN2/4D30, rDEN3D30/31, and rDEN4D30, which is in the clinical trial phase and has more resistance against DENV2 than CYD-TDV does [45,46]. It was found that, after vaccinated with TV003, antibodies of the four DENV serotypes were detected in 91.7% of subjects and that the protective potency against DENV2 was superior to that of CYD-TDV vaccination, with the only adverse reaction presenting a mild rash [47]. More DENV2 attenuated virus components were added to TV005 than to TV003. A phase I clinical trial showed that a single vaccination of TV005 caused a relatively balanced immune response in 90% of vaccinnees while TV003 vaccination only caused immune response in 76% of recipients [46]. TV003 and TV005 induce the most balanced neutralizing antibodies among the five LATVs (TV001–TV005) [27].

TV003/TV005 is very different from CYD-TDV in characteristics due to their different virus particle structure, infectivity, and immunogenicity. For example, CYD results in a higher risk of viremia, lower resistance against DENV2 virus, and lower level of induced immune balance than TV003/TV005 [27].

### 2.3. Live Attenuated Chimeric Tetra-Dengue Vaccines

Scientists used the attenuated virus DENV2 PDK-53 as a genetic backbone to replace its coding sequences with that of DENV1, DENV3, and DENV4; the recombinant RNAs were used to transfect Vero cells to produce a vaccine candidate called DENVax [48]. These tetra-live attenuated virus (TLAV) vaccines are undergoing clinical trials [44]. To investigate whether the vaccination of DENVax in mothers would affect the re-inoculation effects in offspring, AG129 mice were used as the model, and it was found that the vaccination effects of the pups born by PDK53 immunized mothers may be interfered with by their maternal antibodies [23].

In summary, the live attenuated vaccine CYD-TDV has a definitive protectivity in DENV seropositive subjects over 9 years old. The rDEN∆30 vaccine makes up for the low immune balance of CYD-TDV. DENVax is highly immunogenic tolerant and is hard to induce systemic reactions.

## 3. Inactivated Virus Vaccines

Inactivated vaccines are antigenic substances composed of inactivated material from a pathogen (such as virus or bacterium) which can elicit protectivity against the live pathogen [49]. A DENV2 inactivated vaccine named S16803 was developed by formalin inactivation and sucrose centrifugal purification and demonstrated its effectiveness in rhesus monkeys [49,50]. The immunogenicity of the recombinant subunit protein vaccine (R80E) and live attenuated vaccine (DENV2 PDK-50) was compared with the inactivated vaccine S16803 in rhesus monkeys, and it was found that only DENV2 PDK-50 can produce stable titers of antibodies [51]. Tetravalent purified formalin-inactivated virus (TPIV) is an inactivated vaccine containing four inactivated dengue serotypes [52]. Rhesus monkey vaccination initiated with TPIV or tetravalent DNA vaccine (TDNA) and then enhanced with TLAV showed promoted humoral immunity against dengue virus compared with the vaccination using only one type of vaccine [53]. It is reported that the antibody titers of four serotypes of DENV reached a certain height by using TPIV to initiate immunization and then TLAV to enhance immunization [52].

Inactivated dengue virus vaccine uses C, M, E, and NS1 protein components as antigens to stimulate immunity, but composite vaccines arouse better protectivity than single-type vaccines. Compared with live attenuated vaccines, inactivated virus vaccines are safer with no hidden danger of reactivation and better controlled immune balance.

## 4. Recombinant Subunit Vaccines

Recombinant subunit vaccines are antigenic proteins expressed by prokaryotic or eukaryotic cells to stimulate long-lasting protective/therapeutic immune responses [54,55,56]. Expression of the recombinant dengue proteins in *E. coli* is relatively easy, but meanwhile, there are some problems of endotoxin contamination and improper protein folding [57]. The recombinant envelope protein domain III (EDIII) expressed by *E. coli* and purified by metal affinity membrane chromatography was shown to successfully induce antibodies against the four serotypes of dengue in mice [58], and these antibodies also protected lactating mice from infection [59]. Another tetravalent recombinant subunit vaccine combined with alum adjuvant in vaccination produces high titers of antibodies in mice, but it induced only DENV2 antibodies in adult macaques [60,61]. Recombinant dengue proteins fused with a lipoprotein were expressed in a lipid form, thereby eliminating the use of adjuvants, and the viral antigens successfully provoked immune responses against the four serotypes in mice [60,62,63]. In addition, EDIII-P64K was an adjuvant-containing tetravalent dengue vaccine expressing P64K of *Neisseria meningitidis* and EDIII of different DENV serotypes [64]. The mice were immunized with EDIII-P64K three times, and high titers of antibodies against DENV1–3 and low titers of antibodies against DENV4 were produced [65]. Furthermore, a combination of DENV1–2 EDIII and DENV3–4 EDIII linked with a Gly-Ser linker was expressed in *E. coli*, and this vaccine successfully induced immune protection against the four serotypes of DENV in mice. [66]. LTB-scEDIII was a fusion protein expressed by *Saccharomyces cerevisiae,* containing the *E. coli* heat-labile toxin protein B-subunit (LTB) and the synthetic consensus dengue envelope domain III (scEDIII) from the four serotypes. Oral immunization with the intact recombinant yeast cells (rYC) and the cell-free extracts (CFE) was found to stimulate systemic humoral and mucosal immune responses in mice. The titers of neutralizing antibodies in CFE-fed mice were higher than that in rYC-fed mice [67]. 

The most promising subunit vaccine is V180, which consists of a truncated protein DEN-80E expressed in insect cells. V180 vaccination in a low dose successfully induced a high level of immune protection against DENV in mice and rhesus monkeys [68]. It has been reported that V180-immunized rhesus monkeys can be protected from viremia [69]. Sf-9 cells expressing DENV E protein elicited not only specific Th2 responses and weak Th1 responses but also neutralizing antibodies against DENV1-4 [70,71]. Furthermore, recombinant DENV2 NS1 protein expressed with S-2 cells successfully elicited immune protection in mice [12]. A fusion protein containing the DENV consensus domain III (cEDIII) and polymeric immunoglobulin G scaffold (PIGS) was expressed in Chinese ovary hamster cells and transgenic plants, which induced high titer of IgG antibody in mice and showed neutralizing potential against DENV2 [72]. The production of DENV EDIII in transgenic non-nicotine tobacco illustrates the feasibility of plant transgenic vaccine [73], but it had not been tested in any animal models. A single polypeptide chain comprising EDIII of all four serotypes tetra-EDIII (tEDIII) and coenzyme 1 (Co1) was expressed in rice, and the transgenic rice containing 100 μg of tEDIIII-Co1 was fed to mice, presenting strong antigen-specific B and T cell responses [74]. 

Compared with live attenuated vaccines, recombinant subunit vaccines are more likely to trigger balanced immune responses against the four serotypes, reducing the incidence of ADE effect [55,56].

## 5. Viral Vectored Vaccines

Vaccinia virus, adenovirus, and alphavirus vectors have been used as delivery vectors for DENV antigens in vaccine development [11]. It is recorded that prM, E, NS1, and NS2A proteins of DENV4 expressed low efficiency in Cidofovir-resistant vaccinia (WR) strain [75]. Full-length or c-terminal truncated vaccinia virus were recombinated to express DENV E protein to enhance their protectivity [76]. However, the non-attenuated WR strain may bring safety hazards. Immunization of mice with these recombinant vaccines induced only a low level of specific antibodies against E protein [77,78]. Based on the safer modified vaccinia Ankara (MVA) virus, MVA-DENV2–80%E and MVA-DENV4–80%E were then constructed, which can induce high anti-E antibodies in mice, but the former produced low levels of antibodies against DENV2 in rhesus monkeys [79].

Adenoviral vectors show many advantages such as easy gene manipulation, easy detection of gene replication defects, and high level of protein expression [80]. A recombinant replication-defective adenovirus (rAd) expressing the E protein of DENV2 was constructed to vaccinate the mice intraperitoneally, which successfully stimulated DENV2 antibodies and specific T cell immunity in these mice [81,82]. Furthermore, when first immunized with rAd and then boosted with a DNA vaccine expressing EDIII, the mice presented protectivity against DENV2 and DENV4 [83]. The cAdVaxD (1–2) and cAdVaxD (3–4), two divalent complex Adenovirus (cAd) -vectored vaccines expressing prM and E of the DENVs, could induce antibodies against four serotypes and T cell immune protection in rhesus monkeys [84,85].

In addition, the alphavirus-vectored dengue vaccines have high potential. High levels of antigen expression were detected in a single round of vaccination with Venezuelan equine encephalitis virus replicon particles (VRP), and antigen presentation was guaranteed due to the adjuvant activity of VRP and the effect of VRP on dendritic cells (DC) in lymph nodes [86,87]. VRP expressing the M and E proteins of DENV1 induced protective antibodies in cynomolgus monkeys [88]. Immunization with DENV2 VRP in mice produced specific IgG and neutralizing antibodies against DENV2. VRP was constructed to express prM-E and E85; both E85-VRP and prM-E-VRP produced a serotype-specific antibody against EDIII in rhesus monkeys immunization, but E85-VRP developed antibodies faster and with higher titers [89]. The tetravalent E85-VRP dengue vaccine induced a balanced immune response and protectivity against DENV1–4 in monkeys with 2 doses given 6 weeks apart [89]. A tetravalent VRP vaccine induced antibodies at an equivalent level as a monovalent vaccine did after a single immunization, indicating that it could overcome serotype interference [90]. Although the magnitude of the neonatal immune response was lower than that of adult mice, experiments have shown that the VRP vaccine produced a strong protective immunity after a single neonatal immunization [90].

Viral vectored vaccine is still the best way to induce cellular immunity, and it is hopeful to induce stronger humoral responses. Compared with the other viral vectored vaccines, adenoviral vectors are superior in easy genetic manipulation, detection of gene replication defects, and high antigen expression.

## 6. DNA Vaccines

A DNA vaccine is a plasmid containing one or more genes encoding specific antigens, which can be injected in vivo to express antigens and to stimulate immune responses [91]. A DNA vaccine expressing prM and 92% of E protein of DENV2 was used to vaccinate BALB/c mice intradermally, and anti-dengue antibodies were detected in all these mice [91]. When the DNA vaccine expressing E80 (80% of E protein) was compared with the one expressing ME100 (prM and 100% of E protein), the ME100 was found to stimulate antibody production more efficiently in mice [92]. *Aotus nancymae* monkeys were immunized with D1ME100 intradermally and intramuscularly and then boosted at 1 and 5 months post priming; *Aotus* monkeys were partially or completely protected against DENV1 challenge at 6 months post priming [93]. In a human test, D1ME100 showed safe and well-tolerant effects in the first phase of vaccination and the most common side effect was mild pain or tenderness at the injection site. However, the immunogenicity of the vaccine was poor; only 41.6% of the subjects receiving high-dose vaccination produced neutralizing antibodies, and no neutralizing antibody response was detected in the low-dose group [94].

A DNA vaccine expressing DENV2 prM/E fused with the immunostimulatory CpG motif was reported to produce protective immunity against DENV2 and to improve the neutralizing antibody response efficiently compared with the DENV2 prM/E DNA vaccine [95]. Moreover, the DENV2 DNA vaccine expressing a recombinant protein containing DENV2 EDIII and *Escherichia* maltose-binding protein (MBP) was used to immunize mice and was found to be able to elicit neutralizing antibodies in mice [96]. Scientists have evaluated the efficacy of three nonreplicating DENV2 vaccines in rhesus monkeys alone or in combination: DENV2 prM/E DNA vaccine (D), DENV2 EDIII and MBP recombinant fusion protein (R), and purified inactivated virus particles (P). The results showed that DNA vaccine used alone induced a moderate level of neutralizing antibodies while DNA vaccine combined with recombinant proteins induced higher titers of antibodies and neutralization levels. The antibody titers of DR/DR/DR, DP/DP/DP, and R/R/R vaccination were the highest, while the titer of D/D/D vaccination was the lowest. Interestingly, significant immune protection against viremia was observed only in the animals vaccinated with P [97]. 

Some guiding proteins targeting the immune system were used to enhance the protectivity in DNA vaccine development. For example, integrating antigen sequence into lysosomal membrane protein increases the expression of MHC class II antigens, thereby enhancing the production of CD4 T cells and anti-CD4 antigens and ultimately improving the immunogenicity of the DENV2 prM/E DNA vaccine [98]. A DNA vaccine expressing an antigen fused with a single-chain Fv antibody (scFv) specific for the DC endocytic receptor DEC205 could induce a strong immune response to the target antigen [99]. 

The combination of tetravalent DNA vaccine (TVDV), tetravalent purified formalin-inactivated virus (TPIV), and tetra-live attenuated virus (TLAV)-enhanced vaccination strategy was tested in rhesus monkeys, and monkeys immunized with TVDV/TVDV/TLAV were partially protected while TPIV/TLAV immunized monkeys were completely free of viremia [64]. In phase 1 clinical trials, a TVDV with Vaxfectin^®^ (Vical, Boulder, CO, USA) adjuvant was found to trigger anti-dengue T cell IFNγ response with ideal safety [100]. 

DNA vaccines are stable, easy to prepare, low in cost, and suitable for mass production but lack high immunogenicity. Therefore, plasmid modification with highly efficient promoters, alternative delivery strategies, multiple doses, and co-immunization with adjuvants may be the ways to solve this problem [101].

## 7. Conclusions and Future Perspectives

In general, there are five types of vaccines against DENV including live attenuated vaccine, inactivated vaccine, recombinant subunit vaccine, viral vectored vaccine, and DNA vaccine. Among the dengue vaccine candidates in use or in clinical trials (Table 1), live attenuated tetravalent dengue vaccine (CYD-TDV) can stimulate neutralizing antibodies in humans, but the lack of ability to neutralize DENV2 virus limits its use. Immunization of female mice with the live attenuated vaccine DENVax may interfere with the vaccination effect of the born pups. Compared with the live attenuated vaccines, recombinant subunit vaccines are more likely to trigger a balanced immune response against the four serotypes but bring problems of endotoxin contamination and improper protein folding. Adenoviral vectored vaccines show many advantages, such as ease of genetic manipulation, easy detection of gene replication defects, and high levels of protein expression. Alphavirus-vectored dengue vaccines produce a strong protective immunity after a single neonatal immunization in mice. DNA vaccines are stable, low in cost, and easy for mass production; however, the problem of low immunogenicity remains to be solved. In terms of vaccination strategy, the immunization priming and boosting with different vaccine combinations has been used to develop successful protectivity in animals. Furthermore, new vaccine development concepts have been proved to be viable, such as DENV NS1-based and mosquito-based immunization strategies [12]. In addition, studying the immune mechanism of viral components in disease transmission is helpful to break through the bottleneck of vaccine development.

## Figures and Tables

**Table 1:** Dengue vaccine candidates in use or in clinical trials.

Vaccine Type	Name	Strategy	Clinical Trial Phase
Live Attenuated vaccine	CYD-TDV	Replacing the prM/E gene of the YF17D virus with genes of the DENV1–4	Evaluation after part of the license
	TV003/TV005	Attenuation by truncating 30 nucleotides in the 3′ UTR of DENV1, DENV3, DENV4, and a chimeric DENV2/DENV4	Phase III
	DENVax	Replacing the coding sequences of DENV2 PDK-53 attenuated vaccine with that of DENV1, DENV3, and DENV4	Phase III
Inactivated virus	PIV	Purified formalin-inactivated virus and adjuvants	Phase I
Subunit vaccine	V180	A recombinant truncated protein containing DEN-80E	Phase I
DNA vaccine	D1ME100	Recombinant plasmid vector encoding prM/E	Phase I
	TVDV	Recombinant plasmid vector encoding prM/E proteins of DENV1–4	Phase I
Heterologous prime/boost	TLAV Prime/PIV boost and reverse order	Initial immune-boost strategy	Phase I

CYD-TDV: the live attenuated chimeric yellow fever 17D virus-tetravalent dengue vaccine; DENVax: the live attenuated tetravalent dengue vaccine; PIV: the purified formalin-inactivated virus vaccine; TVDV: the tetravalent DNA vaccine; TLAV: the tetra-live attenuated virus vaccine.
